# Murine iPSC-Loaded Scaffold Grafts Improve Bone Regeneration in Critical-Size Bone Defects

**DOI:** 10.3390/ijms25105555

**Published:** 2024-05-20

**Authors:** Franziska Kessler, Kevin Arnke, Benjamin Eggerschwiler, Yvonne Neldner, Sonja Märsmann, Olivier Gröninger, Elisa A. Casanova, Fabienne A. Weber, Matthias A. König, Wendelin J. Stark, Hans-Christoph Pape, Paolo Cinelli, Simon Tiziani

**Affiliations:** 1 Department of Trauma Surgery, University Hospital Zurich, University of Zurich, Rämistrasse 100, 8091 Zurich, Switzerlandelisa.zimmermann@kispi.uzh.ch (E.A.C.); paolo.cinelli@usz.ch (P.C.);; 2 Institute for Chemical and Bioengineering, ETH Zurich, 8093 Zurich, Switzerland; 3 Institute of Laboratory Animal Science, University of Zurich, 8091 Zurich, Switzerland; 4 Ortho Health Munich, 80333 Munich, Germany; 5 Center for Applied Biotechnology and Molecular Medicine (CABMM), University of Zurich, 8057 Zurich, Switzerland

**Keywords:** bone healing, induced pluripotent stem cells, stem cell therapy, osteogenic differentiation, mouse model, critical-size bone defect, musculoskeletal

## Abstract

In certain situations, bones do not heal completely after fracturing. One of these situations is a critical-size bone defect where the bone cannot heal spontaneously. In such a case, complex fracture treatment over a long period of time is required, which carries a relevant risk of complications. The common methods used, such as autologous and allogeneic grafts, do not always lead to successful treatment results. Current approaches to increasing bone formation to bridge the gap include the application of stem cells on the fracture side. While most studies investigated the use of mesenchymal stromal cells, less evidence exists about induced pluripotent stem cells (iPSC). In this study, we investigated the potential of mouse iPSC-loaded scaffolds and decellularized scaffolds containing extracellular matrix from iPSCs for treating critical-size bone defects in a mouse model. In vitro differentiation followed by Alizarin Red staining and quantitative reverse transcription polymerase chain reaction confirmed the osteogenic differentiation potential of the iPSCs lines. Subsequently, an in vivo trial using a mouse model (*n* = 12) for critical-size bone defect was conducted, in which a PLGA/aCaP osteoconductive scaffold was transplanted into the bone defect for 9 weeks. Three groups (each *n* = 4) were defined as (1) osteoconductive scaffold only (control), (2) iPSC-derived extracellular matrix seeded on a scaffold and (3) iPSC seeded on a scaffold. Micro-CT and histological analysis show that iPSCs grafted onto an osteoconductive scaffold followed by induction of osteogenic differentiation resulted in significantly higher bone volume 9 weeks after implantation than an osteoconductive scaffold alone. Transplantation of iPSC-seeded PLGA/aCaP scaffolds may improve bone regeneration in critical-size bone defects in mice.

## 1. Introduction

Critical-size bone defects are characterized by bone loss (e.g., through trauma or infection) that does not heal spontaneously and thus commonly requires surgical intervention [1,2]. Although there are different definitions, gaps greater than 2.5 cm or bone loss of more than 50% of the circumference are generally considered “critical-size” [2]. Current management strategies involve autologous bone grafting, which is the gold standard and believed to be superior to allogenous material [3]. However, harvesting such grafts, most commonly at the iliac crest, is associated with considerable donor site pain and morbidity [1,4,5]. Bone tissue engineering is considered a possible alternative and requires the combination of appropriate cells, growth factors and an adequate biomaterial [6]. The use of ex vivo expanded mesenchymal stromal cells (MSCs) in combination with appropriate scaffolds could be a valuable alternative to autologous bone grafting [7]. MSCs nevertheless consist of a heterogeneous population of cells, and depending on the donor, tissue source and isolation procedure, they exhibit various differentiation potentials toward osteoblasts [8]. Furthermore, the culture conditions used for expanding MSCs are very permissive, and prolonged culture time leads to massive clonal selection [9] and loss of potency [8], resulting in decreased differentiation capacity [10,11]. A new homogenous cell source for the production of bioengineered PLGA/aCaP scaffolds would therefore be of advantage. In this context, induced pluripotent stem cells (iPSCs) represent an attractive alternative to MSCs. They can be generated by reprogramming somatic cells, are pluripotent, can be expanded as a homogenous population and can be cultured under defined conditions. iPSCs have the potential to differentiate into osteoblasts and eventually form bone tissue [11,12]. The biocompatibility of different scaffold materials, such as calcium phosphate cement (CPC), polycaprolactone, borophosphosilicate glass, polyethersulfone, PLGA and hydroxyapatite-coated PLGA of iPSCs in the context of bone and cartilage regeneration have been studied [13,14,15,16,17,18]. An important property of the scaffold is that it should mimic the natural extracellular matrix (ECM) until the cells populating the scaffold synthesize their own ECM [19]. Therefore, the scaffold should have similar properties to natural bone regarding its three-dimensional porous structure, biocompatibility and bioresorbability, and surface texture for appropriate cell migration and proliferation [20]. Bone ECM plays an important role for cell adhesion, proliferation and the regulation of many biological functions like responses to growth factors and differentiation. Bone ECM is necessary for driving the production of new bone by cells of the osteoblast-lineage and the absorption of bone by osteoclasts. In the context of bone regenerative medicine, this knowledge is employed to generate ECM-containing scaffolds by decellularization or for the production of ECM modified scaffolds (for a review, see [21]).

A number of biomimetic materials for bone regeneration have been developed, for instance, several hydrogels, hydrocolloids, collagen-based materials, 3D-printed nanofibers or polymers [22,23,24,25,26]. The advantage of synthetic polymers is that their production is highly reproducible and more controllable compared to natural polymers [27]. Poly (DL-lactic–glycolic acid) (PLGA), a linear copolymer with varying ratios of its monomers glycolic acid and lactic acid, is commonly used for the production of bone scaffolds [27]. PLGA scaffolds can be produced by electrospinning, and due to their high porosity, favor cell adhesion and migration [28]. Their combination with amorphous calcium-phosphate nanoparticles (aCaPs) has been shown to increase surface roughness and thereby facilitate cell adhesion and osteoblast differentiation, which in turn increase the osteoconductive and mechanical properties of the PLGA/aCaP scaffolds [29,30,31,32]. 

To date, the biocompatibility of PLGA/aCaP scaffolds has been extensively tested with mesenchymal stromal cells (MSCs). in various animal models [33,34]. 

Despite the promising results obtained with MSCs [35], the potential of differentiation of iPSCs on PLGA/aCaP scaffold was never tested, neither in vitro nor in vivo. In this study, we therefore aimed for the first time at studying the interaction of iPSCs with PLGA/aCaP scaffolds and at assessing the potential of iPSC-seeded and iPSC-ECM-loaded PLGA/aCaP scaffolds for treating critical-size bone defects in an in vivo mouse model. 

## 2. Results

### 2.1. In Vitro Assessment of Osteogenic Differentiation Capacity of iPSCs

**Figure 1:**
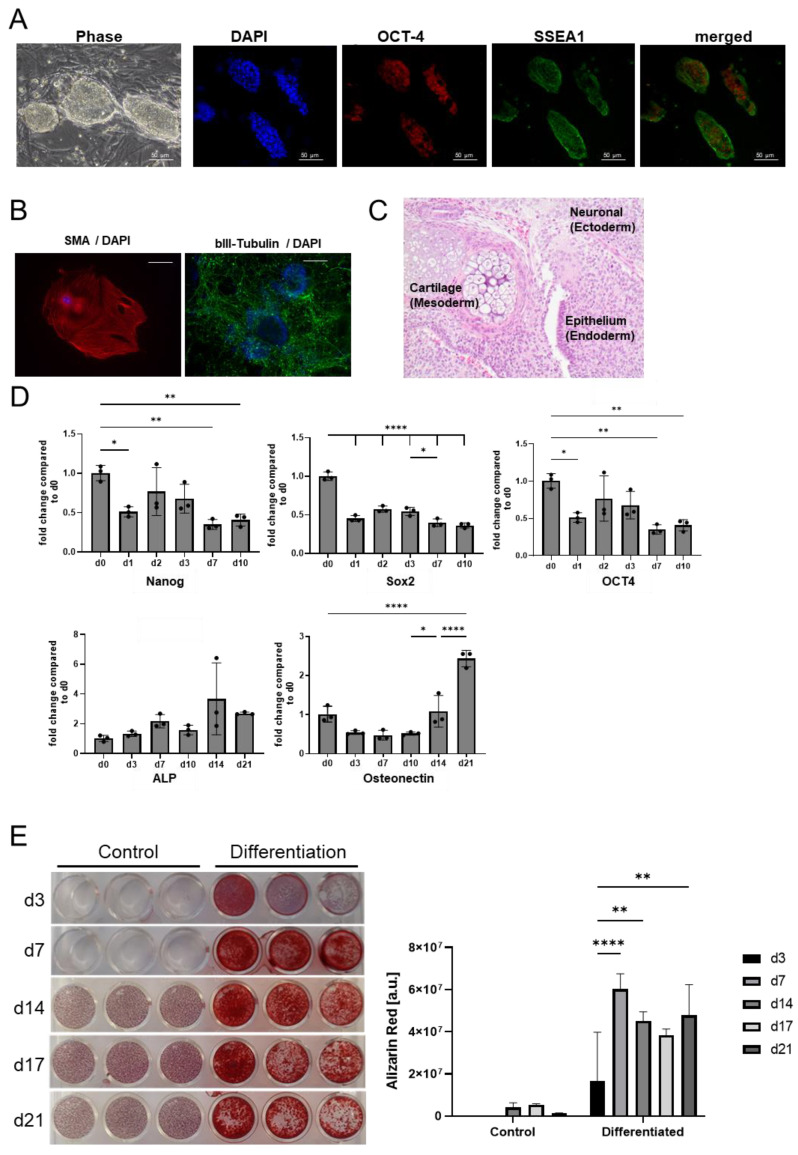
(**A**). Phase-contrast imaging DAPI-staining and immunofluorescence-staining for Oct-4 and SSEA-1 of iPSCs after reprogramming. 40× magnification; scale bar shows 50 µm. (**B**). Anti-SMA/DAPI-staining (mesoderm, left) and anti-bIII-Tubulin (ectoderm, right) with DAPI-staining as part of the teratoma assay (scale bar = 100 µm). (**C**). H&E-staining of the three germ layers (mesoderm, ectoderm and endoderm) in the teratoma assay (40× magnification). (**D**). Results of RTQ-PCR: Relative expression of pluripotency genes (Nanog, Oct-4 and Sox-2) and osteo-specific genes (ALP, osteonectin) over time compared to d0. Mean value and standard deviation of relative expression of pluripotency genes (Nanog, Oct-4 and Sox-2) and osteo-specific genes (ALP, osteonectin). RTQ-PCR was performed in triplicate. Statistical analysis was performed by one-way ANOVA followed by a post hoc Tukey test. Samples were compared to day 0 and the previous time point. *n* = 3. Significance is indicated by * *p* ≤ 0.05. (**E**). Macroscopic images of Alizarin Red staining of the control group and differentiated iPSCs on days 3, 7, 14, 17 and 21 (**left**). Quantification of Alizarin Red staining of the control group and differentiated iPSCs on days 3, 7, 14, 17 and 21 (**right**). The mean value and standard deviation are shown. Staining was performed in triplicate. Statistical analysis was performed by one-way ANOVA followed by a post hoc Tukey test. Significance is indicated by * *p* ≤ 0.05, ** *p* < 0.01, **** *p* < 0.0001.

iPSCs were previously derived from C57BL/6 MEFs [36]. These cells fulfill the pluripotency requirements, being positive for the expression of Oct-4 and SSEA-1 (Figure 1A), as well as being able to differentiate towards derivatives of the three germ layers in vitro (Figure 1B) and in teratoma assays (Figure 1C). In vitro differentiation over 21 days indicated increased expression of the osteogenic markers alkaline phosphatase (ALP) and osteonectin in parallel to a progressive decrease in the expression of the pluripotency genes Nanog, Oct-4 and Sox-2 in the first 10 days (Figure 1D).

In order to better assess the differentiation degree of the cells, we have performed Alizarin Red S staining, which enables us to stain calcium deposits produced by osteoblasts [37,38]. Interestingly, already three days after the induction of differentiation, iPSCs start to deposit calcium. The amount of calcium increased progressively over the first seven days, where a maximum could be reached, and was maintained over the next 21 days of differentiation (Figure 1E).

### 2.2. Scaffold Preparation

**Figure 2:**
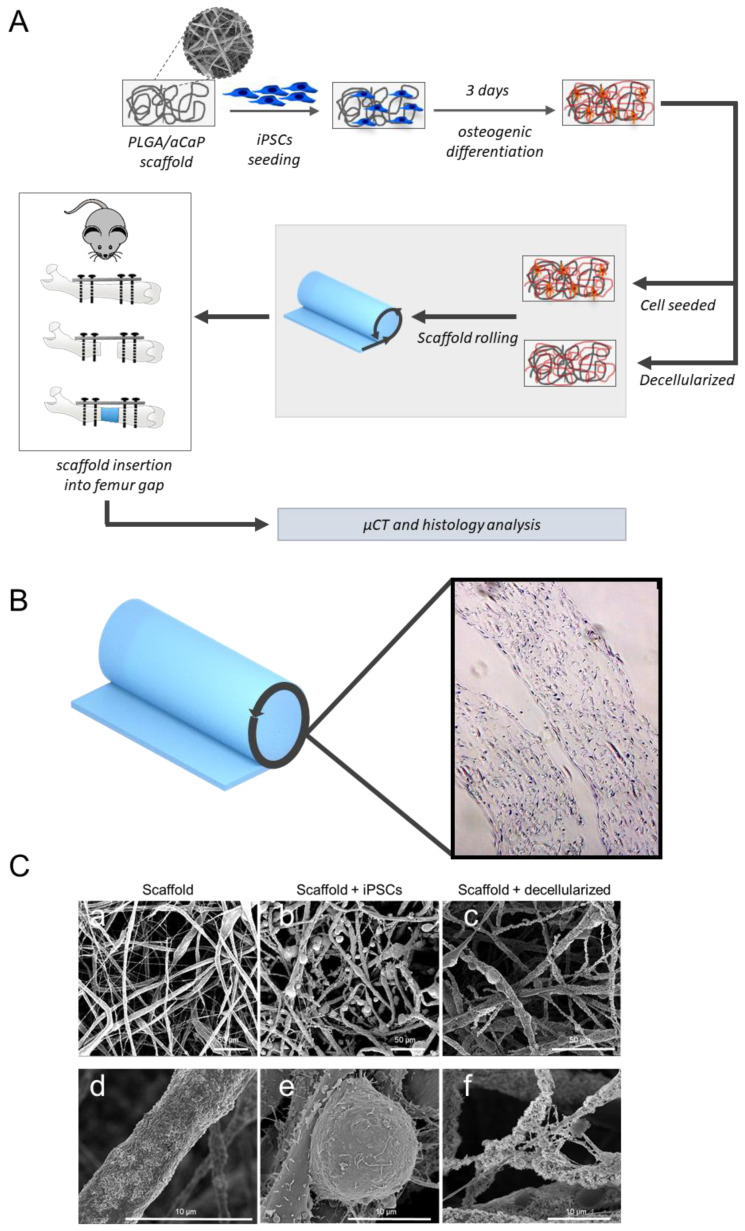
(**A**). Schematic overview of the experimental set-up: iPSCs were seeded on a PLGA/aCaP scaffold, followed by induction of osteogenic differentiation and incubation for 3 days. iPSC-seeded scaffolds were either rolled directly or after chemical decellularization. Performance of a surgical procedure in the mouse with transplantation of the prepared scaffold in the fracture gap. (**B**). Microscopic view of the rolled cell-seeded scaffold and hematoxylin/eosin staining showing the distribution of the cells on the scaffold. (**C**). Representative SEM pictures of scaffold before seeding (**a**,**d**), iPSC-seeded scaffold (**b**,**e**) and decellularized scaffold (**c**,**f**).

We have previously performed a number of studies characterizing the biocompatibility and bone regeneration properties of PLGA/aCaP scaffolds in combination with MSCs [29,30,35,39,40,41]. These scaffolds are designed as bone void fillers with limited tensile properties and are not meant to provide mechanical stability. We decided to employ the same material for assessing the potential use of iPSCs as a cell source for the construction of bioengineered scaffolds. The production of PLGA/aCaP scaffolds by electrospinning offers the possibility of preparing 2D-film-shaped scaffolds, which, upon seeding, allow homogeneous colonization of the surface by the cells and overcome the migration problems typical of 3D-shaped scaffolds. Prior to transferring into the bone gap in the femur, the cell-seeded PLGA/aCaP scaffolds can be rolled to achieve a 3D shape (Figure 2A). Due to the relatively fast differentiation speed of iPSCs (Figure 1E), we decided to pre-differentiate the iPSCs upon seeding on scaffolds for three days before transferring the bioengineered scaffolds into the mouse bone gap. The distribution of the cells in the scaffold upon seeding, three days pre-differentiation, and the rolling of the scaffold were assessed by H&E staining (Figure 2B). We could confirm a uniform distribution of the cells over the entire surface (Figure 2B). For the production of scaffolds loaded with iPSCs-ECM, upon seeding and pre-differentiation for three days, the scaffolds were decellularized by using a chemical treatment with Triton-X-100, which, based on our previous data [35], maintains the best structural integrity of the ECM on PLGA/aCaP. Scaffolds before seeding with iPSCs (Figure 2C, panels a and d), upon seeding (Figure 2C, panels b and e) and after decellularization (Figure 2C, panels c and f) were analyzed by SEM. The homogenous distribution of the cells on the scaffold could be observed, as could the formation of the ECM. 

### 2.3. Mouse Model for Critical-Size Bone Defect

**Figure 3:**
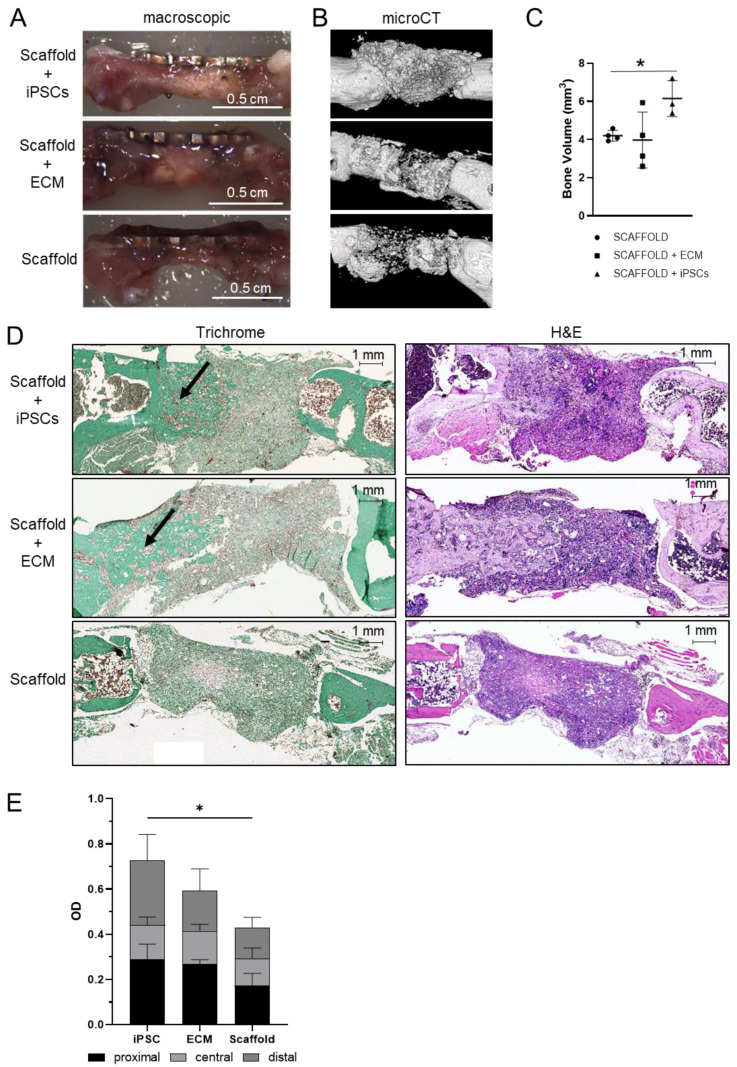
(**A**,**B**). Representative macroscopic images and 3D reconstructions of micro-CT scans of the bone defect 9 weeks after surgery. (**C**). Quantification of bone volume based on micro-CT scans of the defect zone. (**D**). Masson Goldner trichrome (**left**) and H&E staining (**right**) of the bone defect zone. The newly formed bone is marked by an arrow. (**E**). Quantification of collagen production in the defect area based on Masson Goldner Trichome staining (*n* = 4). Areas were divided into proximal, central and distal regions. The mean value and standard deviation are shown. Statistical analysis was performed by one-way ANOVA followed by a post hoc Tukey test. Significance is indicated by * *p* ≤ 0.05. Next to the bone volume, the collagen production was quantified in the newly formed bone, as collagen is a major component of bones (Figure 3E). For this, the fracture region was separated into three different regions (proximal, central, and distal) to ensure a better view of the bone formation, and the amount of collagen was determined by measuring pixels for collagen-specific color in Masson-Goldner trichrome staining. In this histological staining method, collagen is stained turquoise. All three groups showed the largest collagen depositions in the proximal regions and the lowest values in the central regions. Both groups, the iPSC-group and the ECM-group, had similar collagen production in the proximal and central regions, while the scaffold-group showed a reduced production rate. In the distal region, the ECM- and the scaffold-group showed similar levels of collagen, while the iPSC-group had higher levels that were similar to the ones in the proximal region. All regions combined, the iPSC-group and the ECM group produced more collagen in the newly formed bone compared to the group with only scaffold, whereby the iPSC-group reached a significant difference.

We used a mouse model for a femoral segmental critical-size defect to test the regenerative potential of PLGA/aCaP scaffolds seeded with iPSCs or coated with iPSC-derived ECM in vivo. The model consists of a 3.5 mm long segmental bone defect in the mid-shaft of the femur that was stabilized through an internal fixation with a titanium micro-locking plate [35,42,43]. Cell-seeded or decellularized scaffolds, prepared as mentioned in the previous paragraph, were implanted into the bone gaps. Before implantation, the scaffolds were rolled along the longitudinal axis, as shown in Figure 2A. The animals were monitored for nine weeks as described in Materials and Methods. During the experimental period, no animal developed any teratoma formation or any infections. Nine weeks post-surgery, the animals were euthanized, the femurs were isolated (Figure 3A), the micro-locking plates were removed and the samples were fixed in 4% formalin. Samples were analyzed first by micro-CT and, in a second step, embedded in paraffin blocks for further histological analysis. Micro-CT analysis revealed the presence of mineralization within all treatment groups (Figure 3B and Supplementary Figure S1), where the scaffold-only group exhibited a very low mineralization degree. Quantification of the total bone volume of the newly formed bone tissue in the critical-size gap revealed a significant difference between the scaffold-alone and scaffold-IPSC groups (Figure 3C). In the scaffold group, new bone formation was randomly distributed over the gap, but no major connection between endogenous bone and scaffold could be observed (Figure 3A). In contrast, scaffolds seeded with iPSCs or loaded with iPSC-ECM showed new bone formation from the proximal and distal endogenous bone sides, indicating better osseointegration of the scaffolds (Figure 3D,E). These observations could also be confirmed by histological analysis by Masson’s trichrome and H&E staining (Figure 3D). 

## 3. Discussion

Bone exhibits a remarkable regenerative capacity; nevertheless, some injuries result in significant bone loss, preventing natural healing. A number of strategies have been developed to replace diseased bone tissue and promote regeneration, but none of the current techniques has proven ideal for tissue regeneration [44,45,46,47], especially in the treatment of fracture nonunion with bone loss [47,48]. Bone tissue engineering represents an alternative approach to treating bone disorders requiring the replacement of injured or dying cells. The direct use of adult cells, like osteoblasts, has the disadvantages of limited availability, donor site morbidity and restricted differentiation potential [49]. Stem cells represent, therefore, an appealing source of cells for regenerative therapies. In particular, MSCs have gained importance over the past years due to their capacity to differentiate into bone, cartilage, and fat, as well as their potent paracrine anti-inflammatory properties [50,51]. We and others have demonstrated that the combination of MSCs in combination with PLGA/aCaP bone substitutes may be a good alternative to autologous bone grafting [29,35,43]. 

However, the cellular heterogeneity and variable differentiation ability of MSCs represent a strong limitation for their use in therapeutic applications. Even though it is possible to identify subpopulations of MSCs with enhanced osteogenic differentiation potential [8,43], in contrast to pluripotent stem cells, the mechanisms that regulate self-renewal and lineage specification in MSCs are largely unexplored, which makes their cultivation very permissive. Pluripotent stem cells have the ability to undergo self-renewal and the capacity to differentiate into all cells of the body, which makes them very attractive for regenerative medicine. In particular, the possibility to generate iPSCs by reprogramming somatic cells represents a breakthrough, providing a promising strategy to obtain patient-specific stem cells for tissue engineering. In particular, for the treatment of critical-size bone defects, iPSCs represent an interesting cell source. Previous studies have employed iPSCs for treating critical-size defects in mice. Ye et al. have studied the osteogenic differentiation potential of mouse iPSCs overexpressing the transcription factor SATB2 in a mouse critical-size calvarial defect model in combination with silk scaffolds and observed enhanced new bone formation [52]. Other studies have pre-differentiated human iPSCs towards the mesodermal lineage and further differentiated them into chondrocytes. With these cells, cartilaginous organoids were prepared and tested in ectopic and orthotopic bone formation models in immunocompromised mice. The organoids were able to bridge the critical-size long bone defects [53]. A more recent study evaluated the osteogenic ability of iPSCs derived from peripheral blood cells upon differentiation to osteoblasts and following transplantation into a rat critical-size calvarial bone defect model with collagen sponge scaffolds. Also, this study could confirm that iPSC transplantation had bone formations superior to those of the control group with only scaffold [54].

In this study, we aimed at assessing the possibility of using autologous iPSCs in combination with PLGA/aCaP scaffolds for treating bone nonunion in an orthotopic transplantation model. We have used mouse iPSCs and performed a syngeneic transplantation in the C57BL/6 inbred strain with the goal of reducing the rejection risk while at the same time having a functional immune system compared to the classically used immunosuppressed mouse strains. This was important because an increasing number of studies clearly indicate that the immune system plays an extremely important role in the repair of bone defects [55]. Generally, the differentiation protocols used for iPSCs are similar to those developed for ESCs and MSCs [44,56,57]. Osteogenic differentiation of iPSCs can be performed either directly through exposure to osteogenic differentiation medium or, alternatively, in two steps: first differentiation of iPSCs toward MSCs (iPSC-MSCs), followed by a second step of osteogenic differentiation of the iPSCs-MSCs [18]. We have decided to directly differentiate iPSCs and could observe that differentiation was very fast; already after three days of differentiation, it was possible to detect the beginning of calcium deposition in vitro. This is interesting because MSCs under the same differentiation conditions need at least 10–14 days [8]. 

Our data indicate that iPSCs attach and proliferate on PLGA/aCaP scaffolds in a similar way as previously observed for MSCs [35], confirming the biocompatibility of the material. These scaffolds were designed as bone void fillers with limited tensile properties and are not meant to provide mechanical stability but rather to mimic the natural extracellular matrix (ECM) until the cells populating the scaffold synthesize their own ECM and attract endogenous cells.

The orthotopic transplantation indicated a superiority of scaffolds containing iPSCs over scaffolds loaded with iPSC-ECM, and no infection or development of pseudoarthrosis could be observed. Our previous results with MSCs have indicated that both scaffolds seeded with cells or decellularized had similar effects in promoting bone regeneration [35]. Previous studies have also shown that ECM from iPSCs can enhance the osteogenic activity of MSCs [58]. In the current experiments, the cells were only pre-differentiated for 3 days before transplantation, a time window that is probably too short for sufficient deposition of ECM. Interestingly, even though the amount of new bone formed between scaffolds alone and scaffolds loaded with ECM is not significantly different, scaffolds alone exhibit bone formation in the scaffold material but no connection with the endogenous bone, reminiscent of pseudoarthrosis. In contrast, ECM-loaded scaffolds promote new bone ingrowth into the scaffold material, similar to scaffolds containing iPSCs. Further experiments are needed to determine if iPSC-ECM is sufficient to promote bone regeneration.

Even though we did not detect the formation of teratoma during the nine weeks of treatment, the reprogramming protocols we used were based on retroviral transduction. This needs to be optimized either by implementing systems allowing the elimination of the exogenous DNA from the host cell genome after the reprogramming (nonintegrating plasmids) or eventually by employing direct reprogramming to induce osteoblast-like cells [59,60,61].

## 4. Materials and Methods

### 4.1. Culture and Characterization of Mouse iPSCs

The iPSCs used in this experiment were previously generated by Weber and collaborators [36]. Shortly, C57BL/6 mouse embryonic fibroblasts (MEF) were reprogrammed using pMXs retroviral vectors producing murine *Oct4*, *Sox2*, *Klf4*, and *c-Myc* according to Yamanaka’s protocol [62]. The characterization of the pluripotent state of the cell lines employed for the current experiments was previously published by our group [36]. For teratoma assays, 1 × 10^6^ cells were injected subcutaneously into each dorsal flank of NOD/SCID mice. Three weeks after injection, teratomas were dissected and fixed in 4% paraformaldehyde. Sections were stained with hematoxylin/eosin. The Veterinary Office of the Canton of Zurich, Switzerland, approved all animal experiments (ZH 233/2010). For the experiments described in this manuscript, cells were thawed and expanded on mitomycin-C-treated MEFs (CD1 feeder cells). Cells were cultured in iPSC-medium containing Dulbecco’s modified Eagle’s medium (DMEM; PAN, Biotech, Santa Cruz, CA, USA) with 15% fetal bovine serum (FBS; Biowest, Nuaillé, France), 1% 100 × penicillin/streptomycin antibiotic mixture ((5000 U/mL penicillin, 5000 µg/mL streptomycin; Biowest), 1% L-glutamine (200 mM; Sigma Aldrich, Buchs, Switzerland), 0.2% ß-mercaptoethanol (ß-MeEtOH; Biowest), 1% non-essential amino acids (NEAAs; Biowest), 1% sodium pyruvate (Biowest) and 1000 U/mL ESGRO murine Leukemia inhibitory factor (LIF; Merck, Zug, Switzerland).

For immunofluorescence staining, cells were fixed with 4% paraformaldehyde, incubated with primary antibodies against Oct4 (rabbit anti-Oct4, Santa Cruz Biotechnology, Santa Cruz, CA, USA) and SSEA-1 (mouse anti-SSEA-1, Millipore, Merck, Zug, Switzerland). Detection occurred using secondary fluorescence-labeled antibodies (goat anti-rabbit Alexa Fluor 594 and goat anti-mouse Alexa Fluor 488 Molecular Probes). The nuclei of the cells were counterstained with 4′,6-diamidino-2-phenylindole (DAPI) (Roche, Basel, Switzerland).

### 4.2. In Vitro Assessment of Osteogenic Differentiation Capacity

iPSCs (passage 5-10) were seeded on 6-well plates with a density of 200,000 cells/well. Osteogenic differentiation was induced using the StemPro^®^ Osteogenesis Differentiation Kit (Thermo Fisher Scientific, Schlieren, Switzerland) containing StemPro^®^ Osteocyte/Chondrocyte Differentiation Basal Medium and 10% StemPro^®^ Osteogenesis Supplement. Differentiation was running for 21 days; during this time, cells were cultured in differentiation medium and incubated at 37 °C under 95% humidity and 5% CO_2_. The medium was changed every 2 to 4 days, depending on the stage of differentiation. On days 0, 1, 2, 3, 7, 10, 14 and 21, cells were collected for RTQ-PCR analysis. RNA extraction was performed using the RNeasy Mini Kit (Qiagen, Hombrechtikon, Switzerland). The concentration of RNA in the isolated samples was measured with the NanoDrop 2000 Spectrophotometer (Thermo Fisher Scientific, Schlieren, Switzerland), so that 500 ng of total RNA from each sample was reverse transcribed using oligo-dT primers (Invitrogen, Thermo Fisher Scientific, Schlieren, Switzerland) and Superscript III (Invitrogen). Using RTQ-PCR, the expression of six genes related to pluripotency (OCT4, SOX2, ALP, Nanog) and osteogenic differentiation (OCN) was assessed with primer sequences as provided in Table 1. All reactions were performed in triplicate and analyzed using the deltaCT-method.

### 4.3. Alizarin Red Staining

Assessment of calcium deposition as an indicator for differentiation into osteoblasts, was performed by Alizarin Red staining. Undifferentiated iPSCs were seeded in 24-well plates with a differentiation and control section each in triplicate. Control cells were incubated in iPSC-medium for all 21 days of differentiation, whereas the differentiation group was in osteogenic medium (StremPro^®^ Osteogenesis Differentiation Kit, Thermo Fisher Scientific, Schlieren, Switzerland). Depending on the experiment at day 3, 7, 14, 17, and 21, the wells were first washed once with PBS (Kantonsapotheke Zürich, Switzerland) and fixed with 4% formalin (Formafix, Hittnau, Switzerland), followed by staining with the prepared working solution (0.7 g Alizarin Red S (Sigma-Aldrich A5533, Buchs, Switzerland) dissolved in 25 mL tab water). Images of the entire wells were acquired with a Cytation 5 imaging reader (BioTek, Agilent Technologies, Basel, Switzerland), and quantification of the efficiency of differentiation was performed according to [63].

### 4.4. Scaffolds

The PLGA/aCaP scaffolds employed in this study were produced according to Hess et al. [30] (Device: IME EC-CLI, voltage applied: 22 kV to −3 kV, relative humidity: 30%, temperature: 25 °C, feeding rate: 3 mL/h, distance to collector: 15 cm, tip kept in chloroform air stream: 100 mL/min) and clinically approved poly-lactic-co-glycolic acid (PLGA; 85% lactic acid: 15% glycolic acid, Boehringer Ingelheim International, Ingelheim, Germany) and amorphous calcium phosphate nanoparticles (aCaP; Ca/P = 1.5) were incorporated using flame spray synthesis according to Loher et al. [64] and incorporated into the PLGA in a ratio of 60/40 wt (PLGA/aCaP) according to [65]. The surface of the scaffolds was investigated by scanning electron microscopy (SEM, FEI, Nova NanoSEM 450, Hillsboro, OR, USA).

Scaffolds were cut to a size of 1.5 × 2.5 cm, placed on 6-well plates, and incubated overnight at room temperature in a sterilization solution of PBS with 1.3% of amphotericin B (Biowest, Nuaillé, France) and 2% of gentamycin sulfate (Biowest, Nuaillé, France). After the chemical sterilization medium was discarded, the scaffolds were allowed to air dry on a biosafety laminar flow bench before being transferred into a 24-well culture plate for subsequent cell seeding and culture. 

For transplantation in the critical-size mouse, model the scaffolds were seeded with 2.5 × 10^6^ iPSCs directly onto the scaffold with a pipette and, upon attachment, incubated in osteogenic medium (StemPro^®^ Osteogenesis Differentiation Kit, Thermo Fisher Scientific, Schlieren, Switzerland). Scaffolds were incubated for 3 days in osteogenic medium at 37 °C under 95% humidity and 5% CO_2_. For the production of decellularized scaffolds containing iPSC-ECM, decellularization was performed as described in [35]. Decellularization was performed using 3% (vol/vol) Triton X-100 (Roche, Basel, Switzerland) in 50 mM Tris-HCL (pH = 8.3), followed by freezing for 48 h at −80 °C and incubation for 12 h in DNase I solution (Qiagen, Hombrechtikon, Switzerland, final concentration 400 U/mL).

### 4.5. Scanning Electron Microscopy (SEM)

SEM was performed according to [29,35,66]. Samples were fixed with 2.5% glutaraldehyde (Axonlab, Baden, Switzerland) and 2% osmium tetroxide (Sigma Aldrich, Buchs, Switzerland). After dehydration with ascending ethanol gradient solutions (30%, 50%, 70%, 90%, 100%), scaffolds were dried using critical point drying (Tousimis, Rockville, MD, USA). Before SEM analysis, all samples were sputter coated with a 4 nm platinum layer. Images were acquired using scanning electron microscopy (SEM, FEI, Nova NanoSEM 450, Hillsboro, OR, USA).

### 4.6. Animals and Surgical Procedures

In this study, we used twelve C57BL/6 mice (Charles River, Sulzfeld, Germany). The animal housing and the study protocol were approved by the Cantonal Veterinary Office, Zurich (ZH 094/2019) and were in accordance with the Swiss Animal Protection Law and the European Directive 2010/63/EU [67] of the European Parliament and of the Council on the Protection of Animals Used for Scientific Purposes. Animals were randomly assigned to three treatment groups (4 mice per group): (1) only scaffold, (2) scaffold + ECM and (3) scaffold + iPSCs. The surgical procedure was the same for all three experimental groups. Upon rolling, the scaffolds (1.5 × 2.5 cm) were cut into 0.5 cm wide pieces. From each scaffold, we obtained an average of three scaffolds (3.5 mm for insertion into the mouse model. 

Before the initiation of each anesthesia procedure, buprenorphine (Temgesic, Reckitt Benckiser, Wallisellen, Switzerland) was administered subcutaneously at 0.1 mg/kg animal weight as a sedative and preventive analgesic. Anesthesia was administered with a nose mask and induced by inhalation of sevoflurane (Sevorane, Abbott, Baar, Switzerland) at a concentration of 5–8% in 100% oxygen at a flow rate of 200 mL/min. The surgical procedures were performed as previously described [35,42,43]. Subsequently, the right hind leg was shaved and sterilized. We began by making an anterolateral skin incision. Dissection was bluntly carried down to the bone, mobilizing the muscle. Next, a 6-hole internal fixator plate (RISystem, Internal Fixator Plate AO-MouseFix, Davos, Switzerland) was placed on the exposed femur, and the most proximal and most distal screw holes were predrilled, followed by the placement of a locking screw (RISystem AG, Davos, Switzerland) into each of these holes. The transverse 3.5 mm long mid-diaphyseal femoral osteotomy was performed using a Gigli saw (0.22 mm; RISystem AG, Davos, Switzerland) with the help of a drill and saw guide (RISystem AG, Davos, Switzerland). The resulting critical-size bone defect was filled with an equally sized piece of scaffold according to the treatment group. Finally, the wound was closed in layers using single-button sutures. 

For postoperative analgesia, the mice received subcutaneous injections of buprenorphine (0.1 mg/kg, Temgesic, Reckitt Benckiser, Wallisellen, Switzerland) for three days after surgery. After surgery, all animals received adequate care and were monitored for vigilance, pain signs, weight loss and limping three times per day in the first three days, afterwards daily from day 4 to day 7, and finally weekly until the end of the experiment. Nine weeks after surgery, a period sufficient to allow almost complete union of the bone fracture, all mice were sacrificed, and femurs were collected. After removal, the osteosynthesis material was stored in 4% formalin for further analyses. 

### 4.7. MicroCT

The complete femurs were scanned in a micro-computed tomography (micro-CT 40, Scanco Medical AG, Brüttisellen, Switzerland) operated at an energy of 70 kV and an intensity of 114 μA. The scans were executed in high-resolution mode, resulting in a voxel size of 15 μm isotropic. The segmentation of bone was performed by applying a grayscale value threshold to the 3D volume. To measure the amount of regenerated bone in the gap, the center of the closest metallic screw to the scaffold on each side was taken as a reference point, a subvolume was extracted, and the number of voxels corresponding to bone was quantified. In reconstructed images, bone tissue was segmented from the background using a global threshold of 12% of the maximum gray value. The mean of the volumes of bone within each group was calculated and plotted in GraphPad Prism. In one sample (scaffold + iPSCs), two screws were not properly held in the bone, and the scaffold moved out of the gap, inducing inappropriate healing of the bone. This sample was omitted from the μ-CT quantification.

### 4.8. Histological Analysis

For histological analysis, samples were decalcified using USEDECALC (MEDITE) for ca. 5 weeks and embedded in formalin. Sections of 3 µm were cut using a microtome. For hematoxylin and eosin (H&E) staining, the sections were incubated in Mayer’s hematoxylin solution (Artechemis), followed by eosin solution (Morphisto). For Masson Goldner trichrome staining, sections were dipped into Weigert’s Iron Hematoxylin solution (Morphisto), followed by Goldner staining solution (Ponceau-acid fuchsin, Carl-Roth), Phosphortungstic acid-Orange G (Carl-Roth) and Goldner staining solution (Carl-Roth). After dehydration and fixation with mounting medium (Pertex), all H&E and Masson Goldner trichrome sections were stored at room temperature and imaged using a slide scanner (Zeiss Axio Scan Z1). 

Quantitative analysis of the produced collagen amount in the new bone was performed using ImageJ by measuring optical density (OD) according to Arnke et al. [68]. The newly formed bone was separated into three regions: proximal (region towards the body), central and distal (region towards the foot). OD was separately measured for each region. In general, five equal rectangles were used per region to determine the average OD per region.

### 4.9. Statistical Analysis

Statistical analysis was performed using GraphPad Prism Version 9.5.1 (GraphPad Version 10 Software Inc., Boston, MA, USA). Due to the syngeneic transplantation of the iPSCs in an inbred mouse strain, we expect a normal distribution. Data analysis was carried out using a one-way analysis of variance (ANOVA), followed by a post hoc Tukey test. Significance is indicated by * *p* ≤ 0.05.

## 5. Conclusions

In summary, our data indicate that iPSCs can be employed for the production of bioengineered PLGA/aCaP for the treatment of critical-size bone defects. This iPSC-based strategy could be employed to overcome MSC heterogeneity and provide an alternative cell source for bone bioengineering. 

## Figures and Tables

**Table 1:** Primer sequences used for RTQ-PCR.

Genes	Primer Forward (5′ → 3′)	Primer Reverse (5′ → 3′)
Nanog	ACA AGG GTC TGC TAC TGA GAT GC	GGA GAC TTC TTG CAT CTG CTG G
Oct4	GGC GTT CGC TTT GGA AAG GTG TTC	CTC GAA CCA CAT CCT TCT CT
Sox2	TAG AGC TAG ACT CCG GGC GAT GA	TTG CCT TAA ACA AGA CCA CGA AA
Alp	CGC CAG AGT ACG CTC CCG CC	TGT ACC CTG AGA TTC GT
Ocn	CAG CCC CTC AGC AGA CTG AA	GTT GTC AGC CAC CAC CTC CT
Actab	CAT CCA GGC TGT GCT GTC CCT GTA TGC	GAT CTT CAT GGT GCT AGG AGC CAG AGC

## Data Availability

Data will be made available on request.

## Supplementary Materials

The following supporting information can be downloaded at: https://www.mdpi.com/article/10.3390/ijms25105555/s1.

## Abbreviations

aCaP	amorphous calcium-phosphates nanoparticles
ALP	alkaline phosphatase
CD1	cluster of differentiation 1
c-Myc	MYC proto-oncogene
DAPI	4′,6-diamidino-2-phenylindole
DMEM	Dulbecco’s modified Eagle’s medium
e.g.	example given
ECM	extracellular matrix
FBS	fetal bovine serum
iPSC	induced pluripotent stem cell
Klf4	KLF transcription factor 4
LIF	leukemia inhibitory factor
MEF	mouse embryonic fibroblast
MSC	mesenchymal stromal cell
NEAA	non-essential amino acids
Oct4	organic cation/carnitine transporter 4
OD	optical density
PBS	phosphate-buffered saline
PLGA	poly (DL-lactic–glycolic acid)
RNA	ribonucleic acid
RTQ-PCR	real-time quantitative polymerase chaine reaction
SEM	scanning electron microscopy
SMA	smooth musce actin
Sox2	SRY-box transcription factor 2
SSEA-1	stage specific embryonic antigen 1
Tris	tris(hydroxymethyl)aminomethane
vol	volume
