# Supplementary material for: Murine iPSC-Loaded Scaffold Grafts Improve Bone Regeneration in Critical-Size Bone Defects
Source: Int J Mol Sci. 2024 May 20;25(10):5555. doi: 10.3390/ijms25105555 (PMC11121928; doi:10.3390/ijms25105555)
Supplement: Supplementary file 1 [file ijms-25-05555-s001.zip › ijms-2984507-supplementary.pdf]

Supplementary Figure S1:

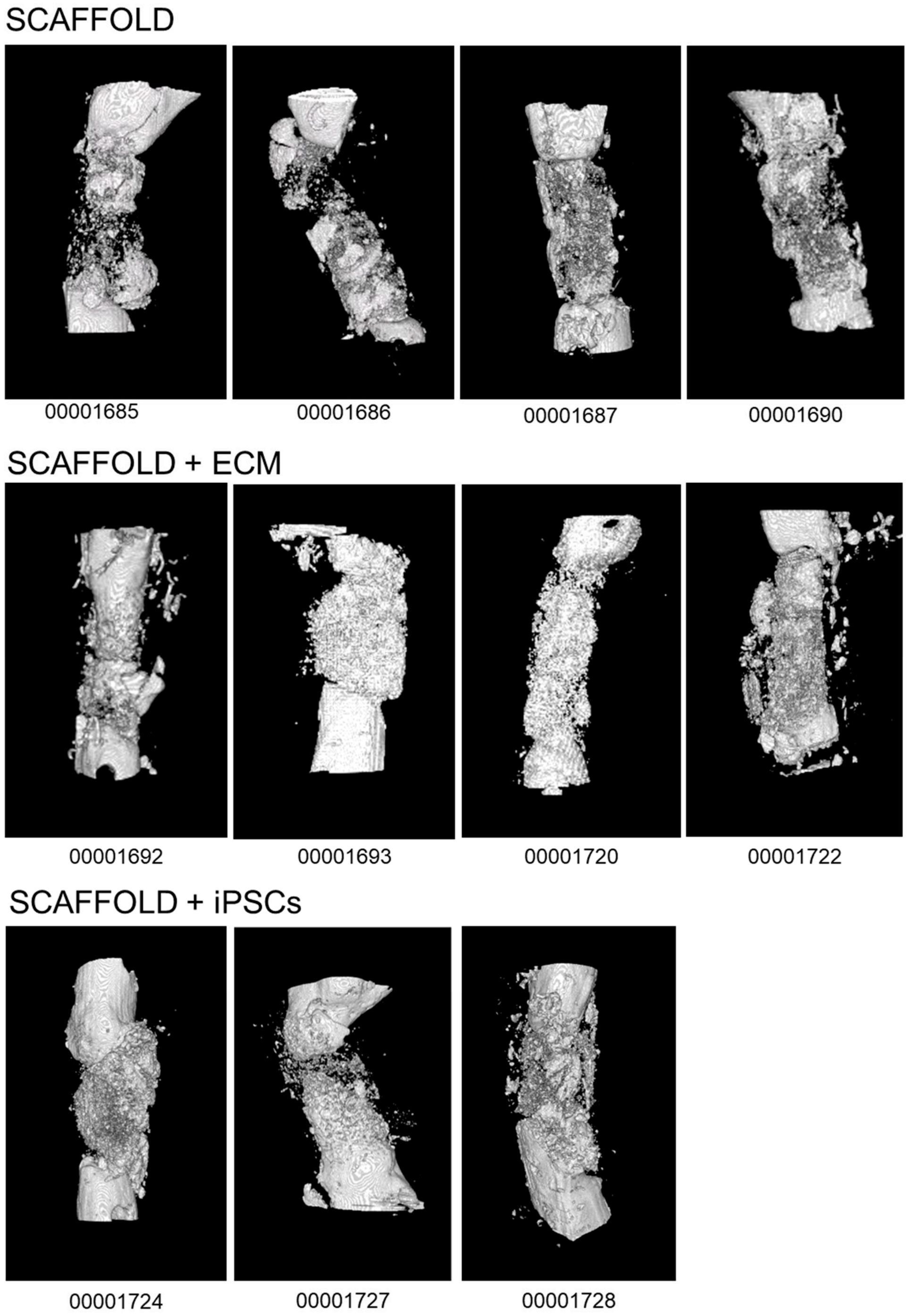

Three-dimensional reconstruction of microcomputed tomography images of all the femurs used in this study, isolated after 9 weeks. Scaffold alone, Scaffold with ECM and scaffold with iPSCs.
